# A new approach for handling missing correlation values for meta‐analytic structural equation modeling: Corboundary R package

**DOI:** 10.1002/cl2.1068

**Published:** 2020-01-31

**Authors:** Soyeon Ahn, John M. Abbamonte

**Affiliations:** ^1^ Department of Educational and Psychological Studies University of Miami Coral Gables Florida

**Keywords:** boundary, meta‐analysis, missing correlation

## Abstract

With increased use of multivariate meta‐analysis in numerous disciplines, where structural relationships among multiple variables are examined, researchers often encounter a particular challenge due to missing information. The current research concerns missing correlations (*r*s) in the correlation matrix of *m* variables (R_*m* × *m*_) and establish more informative and empirical prior distributions for missing *r*s in R_*m* × *m*_. In particular, the method for deriving mathematically/analytically boundaries for missing *r*s in relation to other adjacent *r*s in R_*m* × *m*_, while satisfying conditions for a valid R_*m* × *m*_ (i.e., a symmetric and positive semidefinite correlation matrix containing real numbers between −1 and 1) is first discussed. Then, a user‐defined R package for constructing the empirical distributions of boundaries for *r*s in R_*m* × *m*_ is demonstrated with an example. Furthermore, the applicability of constructing empirical boundaries for *r*s in R_*m* × *m*_ beyond multivariate meta‐analysis is discussed.

## 1 INTRODUCTION

Missing data have long been the bane of data analysists. Part of the reason for the problem that missing data presents is stated in Schafer et al. (2002) review, “Why do missing data create such difficulty in scientific research? Because most data analysis procedures were not designed for them.” As Schafer et al. (2002) highlight, missing data is problematic in a univariate sense. The problem becomes worse when research findings are attempted to be synthesized in a meta‐analytic framework. In these cases, results of many studies are analyzed together, and missingness in one study can propagate throughout the analyses. To make matters worse, in meta‐analysis, missingness can have multiple sources such as from missing studies or missing correlations (Pigott, 1994). Another problem encountered is when authors do not report all intercorrelations between their study variables, due to some associations not being of interest to them (Jak, Oort, Roorda, & Koomen, 2012). This issue may commonly arise when researchers are attempting to study new topics, and wish to conduct an a priori power analysis which requires an estimate of effect size. Some correlations between variables in their model might be reported, but others may be completely missing.

Due to these stated problems, researchers have to deal with the missingness in some way. More simplistic methods involve removing missing cases such as list‐ or pairwise deletion, or using single imputation methods such as mean imputation or cold‐decking (Furlow & Beretvas, 2010). Cold‐decking, and hot‐decking, refer to imputation methodology developed when data were stored on punch cards, and single value imputation was conducted by means of sorting data by different variables and imputing based on the nearest neighbor (Andridge & Little, 2010). Some other studies do not even mention how imputation was conducted (Furlow & Beretvas, 2010). Other more sophisticated univariate weighing procedures have been used as well which include weighing correlations by their variance or using different estimation procedures such as generalized least squares (GLS) to try and capture dependencies between correlations (Furlow & Beretvas, 2005). Results from Furlow and Beretvas (2005) found that applying Fisher's *z* transform (1928) to correlations and weighing them by their inverse variance performed similarly to the more complicated GLS estimation procedure.

## 2 SIMULATION STUDY

In order to test the performance of these variance imputation methods for elements of correlation matrices, a simulation study was conducted in R. A multivariate normal distribution was defined with parameters:

$$\mu =\left(\begin{array}{c} 0\\ 0\\ 0\\ 0 \end{array}\right),\;\Sigma =\left(\begin{array}{cccc} 1 & 0.5 & 0.5 & 0\\ 0.5 & 1 & 0.5 & 0.3\\ 0.5 & 0.5 & 1 & -0.1\\ 0 & 0.3 & -0.1 & 1 \end{array}\right).$$

**Figure 1:**
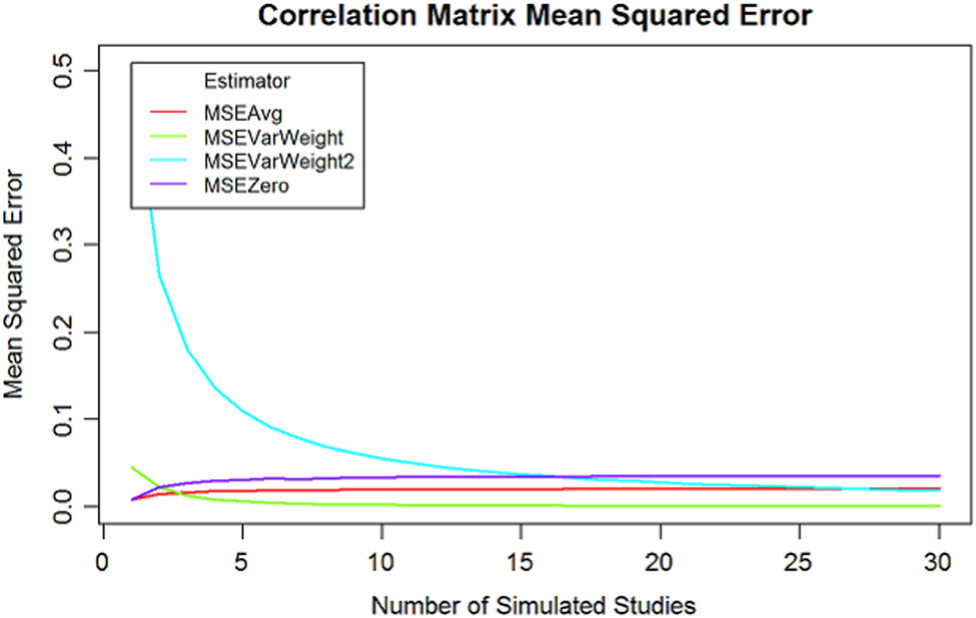
Mean square errors of estimators when number of studies included in meta‐analysis are varied

Next, *n* correlation matrices were estimated from this multivariate distribution. The number of observations used to estimate each of the *n* matrices was normally distributed with a mean of 100 and a standard deviation of 20. Once the *n* matrices were estimated, a missing value was placed into it at random in symmetric locations. Once this was accomplished, the various imputation methods were used to pool the 10 matrices together. Once a pooled matrix was derived, then the mean squared error (MSE) between each location in the lower diagonal of the matrix was computed again Σ. The MSE was then averaged to arrive at an estimate of the performance of the imputation method. This process was repeated 1,000 times. Figure 1 shows the average lower diagonal MSE as the number of matrices estimated is varied from 1 to 30.

MSEAvg refers to imputation based off of the average observed correlation in the missing position. MSEVarWeight first uses Fisher's *z* transformation, and weights the average correlation by the inverse squared standard error of the Fisher transformed correlation (which becomes *N* − 3). MSEVarWeight2 uses the following formula for the variance of a correlation:

$$\mathrm{var}(\rho )=\frac{{\left(1-\rho^{2}\right)}^{2}}{N-1}.$$

The weights were calculated as the reciprocal square of that quantity. Finally MSEZero refers to simply imputing the value 0 for any missing correlation. As can be seen from the graph, the variance weighting procedures tend to outperform the mean and zero imputation methods. These methods offer an advantage by the nature of how the weights are based off of the sample size of the observations which went into estimating the *n*th correlation matrix. Since the sample size was random, larger sample sizes were given more weight, and this led to increased performance.

**Figure 2:**
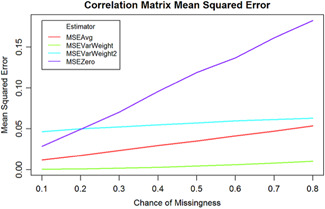
Mean square errors of estimators when the probabilities of missingness are varied

Variance weighting may not always be a feasible approach for researchers, especially when they are study rare phenomenon and are limited by the few studies with small sample sizes. Another factor may also be that correlations are not missing at random. Certain variables may be harder to measure by their nature, and tend to be missing in either how they are reported or collected. To further explore the performance of these estimators, another simulation was conducted. In this simulation, only one correlation from the *n*th estimated correlation matrix was allowed to be missing. This correlation was also in a fixed location. What was varied in this simulation was the probably that the correlation would be missing. Similarly, the correlation matrices were then pooled together, and the MSE was calculated between the fixed location and the population covariance matrix. This was again done 1,000 times for each missing chance from 0.1 to 0.8, and each time 10 correlation matrices were estimated. Figure 2 shows the results.

As can be seen again, inverse‐variance weighting with a Fisher *z* transformed correlation produced the best and most consistent results for various chances of missingness. Computing the average correlation before well, but its performance became much worse as the chance of the correlation being missing increased toward 0.8.

## 3 CORRELATIVE BOUNDARIES

A new method has emerged that may offer some improvements over current univariate approaches for correlation imputation. Researchers doing simulations often need to generate random correlation matrices, and a number of fast methods were developed to accomplish this (Joe, 2006; Lewandowski, Kurowicka, & Joe, 2009). By exploiting naturally occurring interrelationships in correlation matrices, substantial performance gains could be made in algorithmic performance. Numpacharoen and Atsawarungruangkit (2012) and Numpacharoen and Bunwong (2013) developed a novel method of generating random correlation matrices. The approach first takes a correlation matrix, and factors it into a lower triangular matrix *L* by Cholesky decomposition. Therefore, the original matrix is expressed by LL*, with * indicating the transpose of matrix *L*.

The algorithm for performing the Cholesky decomposition is as follows:

Let matrix A be a square, positive definite matrix. The Cholesky decomposition breaks A into a lower triangular matrix *L*, with A = *LL^T^*. The entries in matrix *L* are computed with the following formula (with the Cholesky–Crout algorithm starting in the upper left corner of the matrix *L*, and computing the matrix column by column):

$$L_{j,j}=\sqrt{A_{j,j}-\sum_{k=1}^{j-1}L_{j,k}^{2}}.$$

$$L_{i,j}=\frac{1}{L_{j,j}}\left(A_{i,j}-\sum_{k=1}^{j-1}L_{i,k}L_{j,k}^{T}\right),\;\text{for}\;i>j.$$

Numpacharoen's method then creates another matrix based off *L*, which is called the correlative angle matrix *θ* (technically a reduced form of *θ* with 0 s in the diagonal and upper diagonal entries). This matrix *θ* contains the angles between vectors of variables. The general algorithm to compute *θ* is as follows:

$$\theta_{i,j}=\{\begin{array}{ll} 0, & \text{for}\;i+1\leq j\leq n\\ 1, & \text{for}\;i=1,j=1\\ \cos^{-1}\left(L_{i,j}\right), & \text{for}\;i\geq 2,i=1\\ \sin^{-1}\left(\frac{L_{i,j}}{\prod_{k=1}^{j-1}{\sin(L}_{i,k})}\right),\; & \text{for}\;i=j,2\leq i,j\leq n\\ \cos^{-1}\left(\frac{L_{i,j}}{\prod_{k=1}^{j-1}{\sin(L}_{i,k})}\right), & \text{for}\;2\leq j\leq i-1 \end{array}.$$

Aside from being a fast method of generating correlation matrices, this method also allows one to easily calculate the bounds of multiple correlations compared with other methods developed earlier (Turkay, Epperlein, & Christofides, 2003). By simply moving columns and rows to preserve structure, and setting a position in *θ* to 0 to indicate a maximum correlation and *π* to indicate a minimum correlation, one can obtain the bounds of correlations. A valid correlation matrix has four requires which are being square, having 1 in all positions along the diagonal, all entries being between −1 and 1, and it being positive semidefinite. While at first glance, one may expect that any correlation should have a bound of −1 and 1, but this is not the case. Due to the constraint that the matrix be positive semidefinite (having all eigenvalues ≥0), certain correlations may have a very small degree to which they can vary.

Numpacharoen and Atsawarungruangkit (2012) focus in using these boundary methods were largely to stress test financial portfolios by seeking the minimum and maximum values of correlations. While code in Matlab is available to use Numpacharoen's method for generating random correlation matrices, there does not appear to be an implementation in R, outside the package EvolQG which is used for random matrix generation. We propose extending Numpacharoen's method to be capable of examining the values of missing correlations and fitting distributions on it, and then imputing plausible values based on this correlative boundary.

The current manuscript aims to discuss the method for analytically deriving boundaries for missing correlations in relation to other adjacent correlations in a valid correlation matrix. Then, an R package for constructing the empirical distributions of boundaries for correlations in correlation matrix is demonstrated with an example. Furthermore, the applicability of constructing empirical boundaries for correlations beyond multivariate meta‐analysis is discussed with different examples (such as imputing missing values into a correlation matrix).

## 4 HOW TO USE CORBOUNDARY: A SIMPLE INTRODUCTION

This section will outline how to use the corBoundary package in R to determine the boundaries for a missing correlation (R Development Core Team, 2017).

> library(corBoundary)
> demo_matrix←matrix(c(1, 0.5, 0.5, 0, 0.5, 1, 0.5, 0.3, 0.5, 0.5, 1, −0.1, 0, 0.3, −0.1, 1), nrow = 4, ncol = 4)
> demo_matrix[2, 1]←NA
> demo_matrix[1, 2]←NA
> demo_matrix

$$\text{demo}\_\text{matrix}=\left|\begin{array}{cccc} 1 & \mathrm{NA} & 0.5 & 0\\ \mathrm{NA} & 1 & 0.5 & 0.3\\ 0.5 & 0.5 & 1 & -0.1\\ 0 & 0.3 & -0.1 & 1 \end{array}\right|.$$

This code will load the R package, and create a full correlation matrix. One entry in this matrix is set to missing in both its position below the diagonal and its symmetric position as well.

The first step in the algorithm to find the boundary is to move the missing value to the *N*,*N*_*N −* 1_ position in the matrix, where *N* is the dimension of the correlation matrix.

> demo_matrix←corSwap(demo_matrix, 1, 4)
> demo_matrix←corSwap(demo_matrix, 2, 3)
**>** demo_matrix

$$\text{demo}\_\text{matrix}=\left|\begin{array}{cccc} 1 & -0.1 & 0.3 & 0\\ -0.1 & 1 & 0.5 & 0.5\\ 0.3 & 0.5 & 1 & \mathrm{NA}\\ 0.0 & 0.5 & \mathrm{NA} & 1 \end{array}\right|.$$

These two swaps will move the missing correlation to the correct position while preserving all the correlations in the matrix. With one missing correlation, it should always be possible to move the missing correlation to the *N*,*N*_*N* − 1_ position in two swaps. Once the missing correlation is in the proper location, the correlative angle matrix is solved for.

> angle_matrix←corToAng(demo_matrix)
> angle_matrix

$$\text{angle}\_\text{matrix}=\left|\begin{array}{c} \begin{array}{cccc} 0 & 0 & 0 & 0\\ 1.671 & 0 & 0 & 0\\ 1.266 & 0.987 & 0 & 0\\ 1.571 & 1.044 & \text{NA} & 0 \end{array} \end{array}\right|.$$

The structure of the correlative angle matrix reveals that the missing correlative angle is the last entry in the matrix, and by setting its value between 0 and *π*, the missing correlation can be maximized or minimized, respectively. Once the value has been set at its angle boundaries, it is then converted from a correlative angle matrix back into a correlation matrix, and the correlation in the *N*,*N*_*N* − 1_ position is the respective maximum or minimum correlation.

> angle_matrix[4, 3]←0
> angle_matrix

$$\text{angl}e_{\text{matrix}}=\left|\begin{array}{c} \begin{array}{cccc} 0 & 0 & 0 & 0\\ 1.671 & 0 & 0 & 0\\ 1.266 & 0.987 & 0 & 0\\ 1.571 & 1.044 & 0 & 0 \end{array} \end{array}\right|.$$

>round(angleToCor(bla), 2)[4, 3]
0.95
> angle_matrix[4, 3]←pi
> angle_matrix

$$\text{angle}\_\text{matrix}=\left|\begin{array}{c} \begin{array}{cccc} 0 & 0 & 0 & 0\\ 1.671 & 0 & 0 & 0\\ 1.266 & 0.987 & 0 & 0\\ 1.571 & 1.044 & 3.142 & 0 \end{array} \end{array}\right|.$$

> round(angleToCor(bla), 2)[4, 3]
−0.42

This code produces the two boundaries, in this case 0.42 and 0.95. This is the minimum and maximum values that can be in the first and second variables in the original matrix (or third and fourth variables in the swapped matrix, which are equivalent).

This process can be greatly simplified using the matSolve() which will identify the missing correlation, swap its position, and solve for the boundaries. With this boundary, a point estimate can be calculated (one example would be taking the average) and used in place of the missing correlation.

> matSolve(demo_matrix)
−0.4165129 0.9518664

The corImpute() function can be used to calculate and impute a missing correlation value using a variety of methods, or specific distributions can be defined over the correlative boundary interval and sampled from.

### 4.1 Using corImpute() to create distributions across the correlative interval

Numpacharoen and Atsawarungruangkit (2012) and Numpacharoen and Bunwong (2013) work in the past has been focused on looking at the boundaries of the correlative interval in order to find the smallest and largest correlations that can be achieved. If a researcher wants to impute a plausible value for a missing correlation, it is not obvious what the best choice should be. Any value between the boundaries is valid. One approach would be too simply select a boundary point. Another would be to take the midpoint of the interval (the arithmetic average). More advanced methods could also be used to sample a correlation from the correlative interval. A simple case would be defining a uniform distribution across the interval and sampling from that. This approach might not be the best though, as it is uninformative. The corBoundary package allows researchers to define custom distributions across the correlative boundary. A brief demonstration follows:

> set_seed(12345)
> corImpute(demo_matrix, method = ”custom”, interval_prob = c(.2, .8), interval = c(0, .4, .8, 1))

$$\left|\begin{array}{cccc} 1 & -0.1 & 0.3 & 0\\ -0.1 & 1 & 0.5 & 0.5\\ 0.3 & 0.5 & 1 & 0.918\\ 0 & 0.5 & 0.918 & 1 \end{array}\right|.$$

This code creates a custom discrete distribution on the correlative interval so that there is a 20% chance that a correlation from 0% to 40% of the interval will be uniformly sampled from. Conversely, there is an 80% that a uniformly generated correlation from 80% to 100% of the interval will be sampled. In this case, there is no chance that a correlation between 40% and 80% of the interval will be sampled, although this could easily be added if the researcher desires. The follow two lines lead to the same behavior:> corImpute(demo_matrix, method = “uniform”)

$$\left|\begin{array}{cccc} 1 & -0.1 & 0.3 & 0\\ -0.1 & 1 & 0.5 & 0.5\\ 0.3 & 0.5 & 1 & 0.625\\ 0 & 0.5 & 0.625 & 1 \end{array}\right|.$$

> corImpute(demo_matrix, method = ”custom”, interval_prob = c(1), interval = c(0, 1))

$$\left|\begin{array}{cccc} 1 & -0.1 & 0.3 & 0\\ -0.1 & 1 & 0.5 & 0.5\\ 0.3 & 0.5 & 1 & 0.208\\ 0 & 0.5 & 0.208 & 1 \end{array}\right|.$$

With carefully constructed vectors for both the arguments interval_prob and interval, any discrete distribution can be defined across the correlative interval to the desired accuracy which can be used to approximate any continuous probability distribution.

## CONFLICT OF INTERESTS

The authors declare that there are no conflict of interests.

## Supporting information

Supporting information
