# Supplementary material for: A new approach for handling missing correlation values for meta‐analytic structural equation modeling: Corboundary R package
Source: Campbell Syst Rev. 2020 Jan 31;16(1):e1068. doi: 10.1002/cl2.1068 (PMC8356472; doi:10.1002/cl2.1068)
Supplement: Supplementary file 1 — Supporting information [file CL2-16-e1068-s001.pdf]

Package ‘corBoundary’

July 17, 2017

Type Package

Title Used to calculate boundaries of correlation matrices

Version 0.1.0

Author John Abbamonte

Maintainer John Abbamonte <jma181@miami.edu>

Description Calculates boundaries for correlations in a correlation matrix based on methods developed by Numpacharoen. Based on this boundary, the package can also impute missing correlations using a variety of methods. The package also supports fitting custom distributions to the boundary in order sample an imputed value. This package also contains utility functions for working with correlation matrices.

License MIT

Encoding UTF-8

LazyData true

RoxygenNote 6.0.1

R topics documented:

angleToCor 1
boundary 2
corImpute 3
corSwap 4
corToAng 4
countMiss 5
is.corMat 5
matSolve 6

Index 7

`angleToCor` *Correlative Angle Matrix to Correlation Matrix*

Description

Transform a correlative angle matrix to its correlation matrix form

Usage

```
angleToCor(angMat)
```

Arguments

angMat	A matrix which contains the correlative angles between variables

Value

A correlation matrix corresponding to the input angle matrix

Examples

```
x <- matrix(c(0,0,0,0,1.671,0,0,0,1.266,0.987,0,0,1.571,1.044,0,0), ncol = 4, nrow = 4, byrow = T)
round(angleToCor(x), 2)
```

---

boundary *Correlation Boundary*

Description

Computes the minimum and maximum values for a correlation between two variables in a correlation matrix

Usage

```
boundary(corMat, var1, var2, method = "default")
```

Arguments

corMat	A correlation matrix
var1	A number corresponding to the position in the matrix of the first variable of interest
var2	A number corresponding to the position in the matrix of the second variable of interest
method	"default" computes the bound based of using values of 0 and pi in the correlative angle matrix. "greek" uses a closed form equation to compute.

Value

A vector in which the first element is the minimum correlation and the second element is the maximum correlation

Examples

```
x <- matrix(c(1,.5,.5,0,.5,1,.5,.3,.5,.5,1,-.1,0,.3,-.1,1), nrow = 4, ncol = 4)
x[4,3] <- NA
x[3,4] <- NA
boundary(x, 4, 3)
```

corImpute *Imputes missing correlation*

Description

Recursively imputes the value of missing correlations

Usage

```
corImpute(corMat, method = "average", interval_prob = NA, interval = NA)
```

Arguments

`corMat`	A matrix which contains the correlations between variables
`method`	"average" uses the midpoint (average) of the correlative bound. "lbound" uses the left boundary (closest to -1) of the correlation. "rbound" uses the right boundary (closest to 1) of the correlation. "min" uses the smallest boundary in terms of absolute value. "max" uses the largest boundary in terms of absolute value. "custom" allows for the specification of a specific discrete distribution across the boundary interval to be specified.
`interval_prob`	A vector used with method = "custom". Specifies the probability of sampling from a specific slice of the boundary interval. All elements of the vector must add to 1.
`interval`	A vector that specifies the section of the boundary interval which is to be sampled from. For each probability in "interval_prob" two points must be specified. Points are specified as decimal values which correspond to the proportion of the interval in which to be sample from. For example, c(.5, .8) would sample from 50% to 80% of the boundary interval with a probability specified with the associated "interval_prob".

Value

A correlation matrix with all possible missing values recursively imputed using specific method

Examples

```
x <- matrix(c(1,.5,.5,0,.5,1,.5,.3,.5,.5,1,-.1,0,.3,-.1,1), nrow = 4, ncol = 4)
x[4,3] <- NA
x[3,4] <- NA
corImpute(x)
corImpute(x, method="lbound")
corImpute(x, method="custom", interval_prob=c(.2, .8), interval=c(0, .4, .8, 1))
```

corSwap — *Swaps variables in a correlation matrix*

Description

Moves two variables in a correlation matrix while preserving the structure of the matrix

Usage

```
corSwap(corMat, i, j)
```

Arguments

`corMat`	A correlation matrix
`i`	The position of the first variable to swap
`j`	The position of the second variable to swap

Value

The swapped correlation matrix

Examples

```
x <- matrix(c(1,.5,.5,0,.5,1,.5,.3,.5,.5,1,-.1,0,.3,-.1,1), nrow = 4, ncol = 4)
corswap(x, 1, 4)
```

corToAng — *Correlation Matrix to Correlative Angle Matrix*

Description

Transform a correlation matrix to its correlative angle matrix form

Usage

```
corToAng(corMat)
```

Arguments

`corMat`	A matrix which contains the correlations between variables

Value

A correlative angle matrix corresponding to the input correlation matrix

Examples

```
x <- matrix(c(1,.5,.5,0,.5,1,.5,.3,.5,.5,1,-.1,0,.3,-.1,1), nrow = 4, ncol = 4)
x[4,3] <- NA
x[3,4] <- NA
y <- corToAng(x)
y[4,3] <- 0
round(angleToCor(y), 2)
```

countMiss — *Count missing values*

Description

Counts the number of missing correlations in a correlation matrix (lower triangular)

Usage

```
countMiss(corMat)
```

Arguments

corMat	A correlation matrix

Value

The number of missing correlations

Examples

```
x <- matrix(c(1,.5,.5,0,.5,1,.5,.3,.5,.5,1,-.1,0,.3,-.1,1), nrow = 4, ncol = 4)
x[4,3] <- NA
x[3,4] <- NA
countMiss(x)
```

is.corMat — *Check for valid correlation matrix*

Description

Determines whether a matrix is a valid correlation matrix

Usage

```
is.corMat(corMat, epsilon = 1e-05)
```

Arguments

corMat	A matrix which contains the correlations between variables
epsilon	Numeric treshhold for checking whether a correlation or eigenvalue is out of bounds

Value

TRUE if the correlation matrix is valid, FALSE otherwise

Examples

```
x <- matrix(c(1,.5,.5,0,.5,1,.5,.3,.5,.5,1,-.1,0,.3,-.1,1), nrow = 4, ncol = 4)
is.corMat(x)
x[1,1] <- 5
is.corMat(x)
```

matSolve *Solves for the boundary of one missing correlation*

Description

Automatically detects and returns the bondary of one missing correlation in a correlation matrix

Usage

```
matSolve(corMat)
```

Arguments

corMat	A correlation matrix with one missing correlation

Value

A vector in which the first element is the minimum correlation and the second element is the maximum correlation

Examples

```
x <- matrix(c(1,.5,.5,0,.5,1,.5,.3,.5,.5,1,-.1,0,.3,-.1,1), nrow = 4, ncol = 4)
x[4,3] <- NA
x[3,4] <- NA
matSolve(x)
```

Index

angleToCor, 1

boundary, 2

corImpute, 3
corSwap, 4
corToAng, 4
countMiss, 5

is.corMat, 5

matSolve, 6
